# Loss of CHTF18–RFC2/5 leads to replicative gaps and sensitivity to PARP inhibitors

**DOI:** 10.1093/narcan/zcag013

**Published:** 2026-06-03

**Authors:** Lauryn Buckley-Benbow, Meryem Ozgencil, Alessia Tardocchi, Alessandro Agnarelli, Roberto Bellelli

**Affiliations:** Centre for Cancer Cell & Molecular Biology, Barts Cancer Institute, Queen Mary University of London, Charterhouse Square EC1M 6BQ, London, United Kingdom; Centre for Cancer Cell & Molecular Biology, Barts Cancer Institute, Queen Mary University of London, Charterhouse Square EC1M 6BQ, London, United Kingdom; Centre for Cancer Cell & Molecular Biology, Barts Cancer Institute, Queen Mary University of London, Charterhouse Square EC1M 6BQ, London, United Kingdom; Dipartimento di Medicina Molecolare e Biotecnologie Mediche, Università Federico II, 80131 Napoli, Italy; Centre for Cancer Cell & Molecular Biology, Barts Cancer Institute, Queen Mary University of London, Charterhouse Square EC1M 6BQ, London, United Kingdom; Centre for Cancer Cell & Molecular Biology, Barts Cancer Institute, Queen Mary University of London, Charterhouse Square EC1M 6BQ, London, United Kingdom

## Abstract

The use of PARP inhibitors (PARPi) has profoundly changed the treatment of BRCA1/BRCA2-mutated cancers. Despite this, acquired resistance to PARPi has become a major challenge in the clinic. Hence, a more detailed understanding of the mechanisms underlying PARPi sensitivity is crucially needed. Here, we show that loss of the alternative clamp loader complex CHTF18–RFC2/5 leads to a remarkable sensitization to PARPi. Loss of CHTF18 is not associated with defective RAD51 foci formation excluding a defect in homologous recombination. On the contrary, treatment with PARPi triggers replicative gap accumulation in CHTF18 knockout (KO) cells. By performing transient silencing experiments, we highlight PARP1–PARP2 trapping at replicative gaps as a major determinant of sensitivity to these compounds. Crucially, loss of 53BP1 does not rescue PARPi sensitivity in CHTF18 KO cells, outlining Polε and the CHTF18–RFC2/5 complex as potential novel targets for cancer therapeutics.

## Graphical Abstract

**Graphical Abstract:**
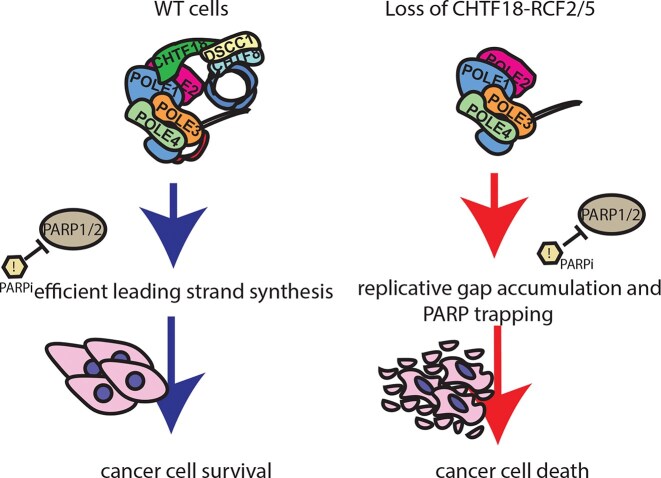

## Introduction

The maintenance of genome stability requires efficient and accurate DNA replication and dysfunction of this process is associated with genetic instability and cancer [1]. On the other hand, by hijacking the replication and repair machineries, cancer cells can become dependent on specific DNA repair mechanisms for survival [2]; the paradigmatic example of this concept is the synthetic lethality between loss of BRCA1/BRCA2—factors critical for homologous recombination (HR)—and PARP inhibitors (PARPi) [3]. Despite two decades of research, the mechanism underlying sensitization of cancer cells to PARPi in cancer remains debated [4]. Recently, replicative gap accumulation emerged as a crucial driver of sensitization to PARPi in a BRCA-mutant background [5]. The sources of replicative gaps in this genetic context remain however unclear [4]. Importantly, while initially effective in HR-deficient cancers, treatment with PARPi is associated with primary and secondary resistance, a growing concern in the clinic [6].

DNA replication is performed by a multiprotein complex known as the replisome. At the heart of this machinery is DNA polymerase epsilon (Polε) a 4 subunits complex (POLE1–POLE4) required for initiation of DNA replication and leading strand DNA synthesis [7]. We recently revealed the genetic and biochemical basis of processive leading strand synthesis by Polε. We highlighted the existence of two tiers of regulation of Polε processivity, relying of Proliferating Cell Nuclear Antigen (PCNA) loading at leading strands by the CHTF18–RFC2/5 complex and double-stranded DNA (dsDNA) binding by the POLE3–POLE4 subunits. Loss of both these tiers is incompatible with efficient DNA synthesis and cell viability [8]. Importantly, we and others have previously shown that deletion of POLE3–POLE4 leads to BRCA1-independent sensitization to PARPi [9, 10]; the mechanism behind this phenomenon remained however uncharacterized.

Here, we show that loss of the Polε processivity factor CHTF18 sensitizes cancer cells to PARPi in an HR-independent manner, similarly to POLE3–POLE4 KO. Transient silencing of PARP1 and PARP2 reduces this effect underlining the role of PARP trapping in this phenomenon. Finally, sensitization to PARPi is resistant to loss of 53BP1, highlighting Polε and the CHTF18–RFC2/5 complex as novel targets for cancer therapy.

## Materials and methods

### Cell lines

The human HeLa TRex Flip In (HTF) cell line was originally obtained from the Boulton lab (Francis Crick Institute). Parental HTF cell lines and their derivative knockout (KO) for CHTF18, POLE3, POLE4, and PARP were grown in Dulbecco’s modified Eagle’s medium (Invitrogen) supplemented with 10% Fetal Bovine Serum (FBS) and 1% penicillin–streptomycin.

## Method details

### Generation of CRISPR knockout cell lines

CRISPR gRNA against PARP1 and PARP2 were cloned into lenti-CRISPR V2-Puro. HTF, HTF POLE3 KO, and HTF POLE4 KO cells were seeded in 10 cm dishes and transfected 24 h later with lenti-CRISPR V2-Puro plasmids using Lipofectamine 2000. Forty-eight hours post-transfection, cells were selected in media containing 1 μg/ml puromycin. Subsequently, cells were seeded at as single cells in 96-well plates and grown for 7–10 days to allow single colonies to form. Single clones were expanded and screened for loss of PARP1 or PARP2 by western blotting.

### Western blot analysis of cell lysates

Cells were lysed in a buffer containing 50 mM HEPES (pH 7.5), 1% (vol/vol) Triton X-100, 150 mM NaCl, and 5 mM Ethylene Glycol Tetraacetic Acid (EGTA), with Protease and Phosphatase inhibitors (ROCHE). Lysates were clarified by centrifugation (13.000 rpm 15 min at 4°C), and protein concentration was estimated by BRADFORD assay (SIGMA). Equal amounts of proteins were loaded on NuPAGE 4%–12% Bis–Tris gels and transferred onto nitrocellulose membrane (Amersham). Membranes were blocked in 5% milk in PBST (PBS–Tween 0.1%) and incubated with primary antibodies and Horseradish Peroxidase (HRP)-conjugated secondary antibodies.

### siRNA transfections

Cells were grown to 40%–50% confluency and transfected with 20 nM small interfering RNAs (siRNAs) against human POLE3, POLE4, CHTF18, REV1, PRIMPOL, BRCA1, and 53BP1 or a negative control (siRNAs listed below) using Lipofectamine RNAiMAX (Thermo Fisher) according to manufacturer’s instructions. siRNAs and lipofectamine were initially diluted in Opti-MEM medium and mixed after 5-min incubation. After an additional 15 min, the transfection mix was directly added to the cells. Forty-eight hours later, cells were seeded for colony forming assays or pellets were collected for western blot.

**Table:** 

siRNA	Reference	Manufacturer
siNT (non-targeting)	D-001810-10-05	Dharmacon/Horizon
siPOLE3	L-008460-01-0005	Dharmacon/Horizon
siPOLE4	L-009850-02-0005	Dharmacon/Horizon
siCHTF18	L-013915-00-0005	Dharmacon/Horizon
siREV1	s28167	Thermo Fisher
siPRIMPOL	GAGGAAACCGUUGUCCUCAGUGUAU	Merck–Sigma–Aldrich, custom synthesized
siBRCA1	L-003461-00-0005	Dharmacon/Horizon
si53BP1		Sigma–Aldrich

### Colony forming assays

For clonogenic survival assays, 500 (or 1000 for CHTF18 KO) cells were seeded per well in 6-well plate format in technical triplicate. Olaparib, AZD6738, Prexasertib, NU7441, HU, or ionizing radiation (IR) were added at different concentrations/doses the subsequent day. Cells were then grown for a further 7–10 days. Surviving colonies were fixed and stained with a solution containing crystal violet, formaldehyde, and methanol then imaged and analysed using imageJ. Treated cells were normalized to untreated samples.

### IncuCyte live cell imaging

To investigate cell proliferation (measured as confluency), cells were transfected with the indicated siRNAs and seeded, after 24 h, at 1000 cells/well in 96-well plates. Cells were left to adhere in the incubator for additional ~18 h. Medium was then replaced with fresh one and plates were incubated in an IncuCyte^®^ S3 Live-Cell Analysis System (Sartorius) at 37°C for 120 h. Cell confluency was calculated by image-based measurements of cell growth using the IncuCyte Analysis Software Version 2023A (Sartorius).

### Immunofluorescence staining

HTF wild type (WT) and CHTF18 KO cells were grown on glass coverslips and treated with Olaparib (0.5 μM) for 24 h or IR for 2 h before fixation in 4% paraformaldehyde (10 min at RT). When described cells were pre-extracted with Cytoskeleton (CSK) buffer containing 0.5% Triton for 10 min on ice before Paraformaldehyde (PFA) fixation at RT. After 3× washes in phosphate-buffered saline (PBS), cells were permeabilized with 0.5% Triton–PBS for 10 min and incubated with primary antibodies for 1 h at RT. Coverslips were then washed three times with PBS and incubated with secondary antibodies for 1 h at RT in the dark. After three washes in PBS, coverslips were incubated in 0.2µg/ml DAPI for 2 min at RT, washed, air-dried, and finally mounted onto glass slides using ProLong Gold Antifade Mountant (Invitrogen). Images were acquired with a Nikon Eclipse Ti-E inverted microscope using 63× objective lens. pS33 Replication Protein A (RPA), RPA, and RAD51 foci quantification was performed using ImageJ. Cell nuclei were defined by thresholding the DAPI signal and the regions of interest were saved to the ImageJ ROI manager. The pS33 RPA, RPA, and RAD51 signal was smoothed with the ‘Smooth’ function, and the noise tolerance was adjusted using the ‘Find maxima’ command to identify the pS33 RPA, RPA, and RAD51 foci. Foci for each pre-selected ROI were automatically counted, and results were presented as the sum of all pixels in the region on the ROI (RawIntDen). Since the pixel value is 255, the sum of all pixels was divided by 255 to quantify the number of pS33 RPA, RPA, and RAD51 foci in the defined nuclear region.

### DNA fiber assay and S1 nuclease assay

Briefly, cells of the indicated genotypes were pulse labelled with 20 μM CldU for 30 min and subsequently pulse labelled with 200 μM IdU for 30 min. Cells were trypsinized, washed in PBS, counted, and resuspended at a concentration of 7 × 10^5^ in PBS. 2.5 μl of cell suspension were spotted on clean glass slides and lysed with 7.5 μl of 0.5% sodium dodecyl sulphate in 200 mM Tris–HCL (pH 7.4), 50 mM ethylenediaminetetraacetic acid (10 min, RT). Slides were tilted (15° to horizontal), allowing a stream of DNA to run slowly down the slide, air-dried, and then fixed in methanol/acetic acid (3:1) for 15 min at RT. Acid-treated slides (45 min RT) were blocked in 1% bovine serum albumin/PBS for 45 min at RT and incubated with rat anti-BrdU monoclonal antibody (1:1000 overnight; Abcam) and mouse anti-BrdU monoclonal antibody (1:500 1h RT; Becton Dickinson). After three washes in PBS, slides were incubated with a mixture of Alexa Fluor 488 rabbit anti-mouse and Alexa Fluor 594 goat anti-rat antibodies (1:500 RT; Invitrogen) for 45 min at RT and mounted in PBS/Glycerol 1:1. Fibers images were acquired with a Nikon Eclipse Ti-E inverted microscope using 63× objective lens and quantification was performed using ImageJ. S1 nuclease assay was performed as described in [11] with the exception than immunofluorescence fiber staining was performed as described above.

### Quantification and statistical analysis

All statistical analysis were performed using GraphPad Prism 9 software. Statistical details for the experiments are provided in the figure legends. *P *< .05 was considered to be significant and classified by asterisks: *P < .05, **P < .01, ***P < .001, and ****P < .0001.

## Results

### Loss of CHTF18 leads to increased sensitivity to ATR–CHK1 and PARP inhibitors

**Figure 1.**
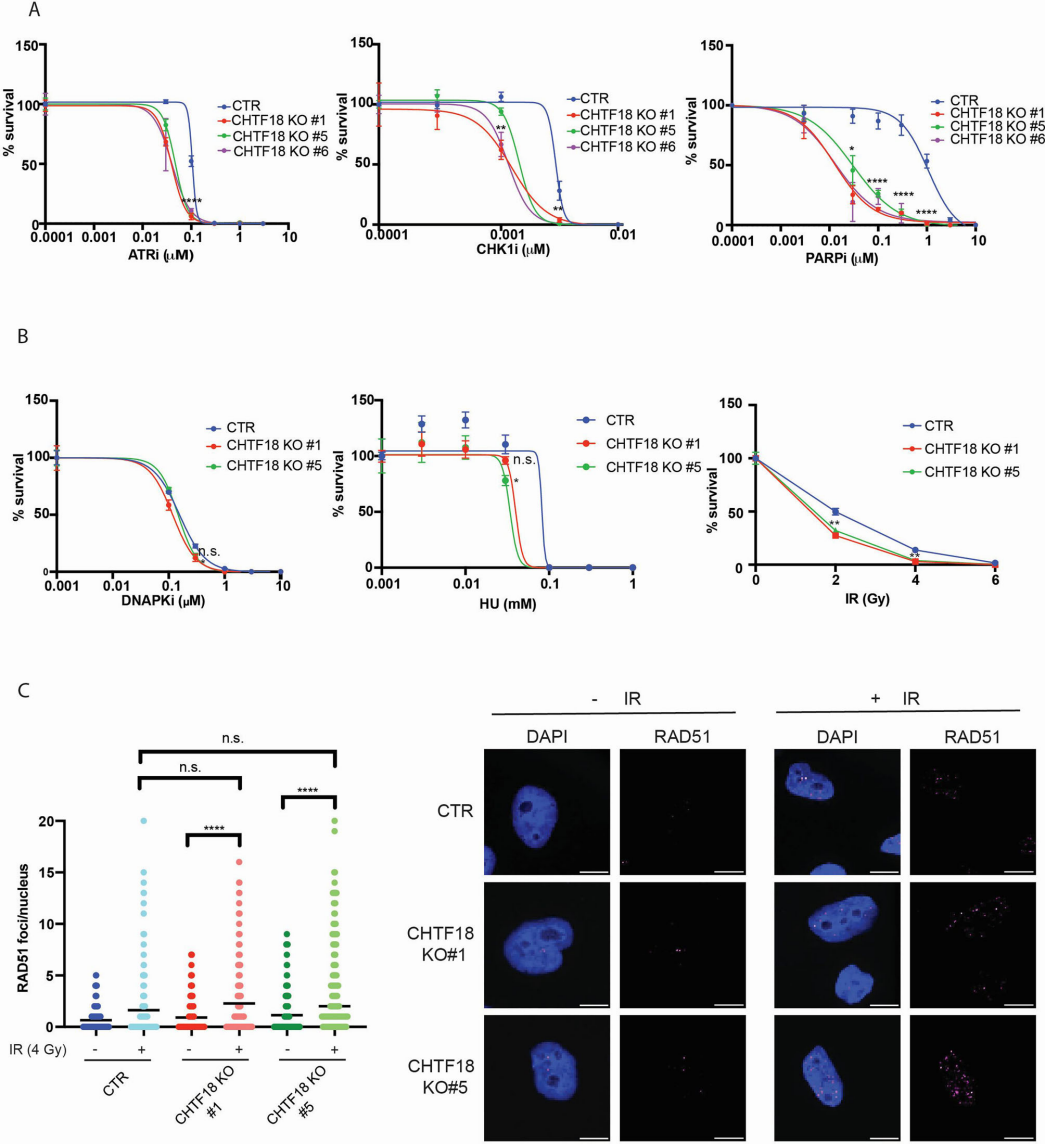
CHTF18 KO cells are hyper-sensitive to ATR, CHK1, and PARPi. (**A**) Quantification of clonogenic survival of CHTF18 KO cells treated or not with increased concentration of AZD6738 (ATRi), AZD7762 (CHK1i), and Olaparib (PARPi). Results were obtained from three independent biological experiments. (**B**) Quantification of clonogenic survival of CHTF18 KO cells treated or not with increasing concentration of NU7441 (DNAPKi), Hydroxyurea, and IR. Results were obtained from three independent biological experiments. (**C**) Left: Bar-graphs showing the number of RAD51 foci/nucleus in the indicated cell lines treated or not with 4 Gy IR. One hundred fifty to two hundred cells were analysed for condition. Right: Representative pictures from immunofluorescence staining for RAD51 in the indicated cell lines treated or not with 4 Gy IR; unpaired *t*-test analysis, ****P < .0001.

We and others have previously shown that loss of POLE3–POLE4 sensitizes cancer cells to ATRi and PARPi in a BRCA1-independent manner [9, 10]. More recently, we have shown that POLE3–POLE4 increase Polε processivity by promoting engagement of Polε with nascent dsDNA [8]. Importantly, in a parallel pathway, the CHTF18–RFC2/5 complex loads PCNA at leading strands to support full Polε processivity. Consistently with this, loss of POLE3–POLE4 is synthetic lethal with genetic deletion of components of the CHTF18–RFC2/5 complex [8]. The increased sensitivity to PARPi in the POLE3–POLE4 KO background and the role of POLE3–POLE4 in promoting Polε-dependent DNA synthesis suggests that loss of Polε processivity might be causing sensitivity to PARPi. In order to validate this possibility, we tested sensitivity of CHTF18 KO cells to a variety of DNA damaging agents and DDR inhibitors. Consistently with our hypothesis, and similarly to POLE3–POLE4 null cells, CHTF18 KO cells showed increased sensitivity to ATR and CHK1 inhibitors (Fig. 1A). More importantly, loss of CHTF18 was associated to a remarkable sensitivity to Olaparib, suggesting that loss of PCNA loading at leading strand leads to increased sensitivity to PARPi (Fig. 1A). Consistently with a PARP-specific vulnerability, CHTF18 KO cells were also hypersensitive to other PARPi such as Veliparib and Talazoparib with lower and higher trapping potency, respectively (Supplementary Fig. S1A). CHTF18 KO cells were not hypersensitive to DNAPKi, excluding unspecific sensitivity to DDR inhibitors and were not sensitive to replication stress inducing agents, such as hydroxyurea and Mitomycin C nor G4 stabilizers (Pyridostatin) or IR, pointing to high specificity (Fig. 1B and Supplementary Fig. S1B). Finally, transient silencing of CHTF18 by siRNA led to increased sensitivity to PARPi in eHAP cells, excluding a cell line-specific phenomenon (Supplementary Fig. S1C).

Increased sensitivity to PARPi is frequently associated with loss of HR-dependent repair, as exemplified by the extreme sensitivity of BRCA1–BRCA2 null cells to PARPi [3]. In order to exclude a possible role of CHTF18 in HR, we then subjected CHTF18 WT and KO cells to treatment with 4 Gy of IR and analysed chromatin recruitment of RAD51 as a surrogate marker of HR proficiency. Strikingly and consistently with an HR-independent mechanism of sensitization to PARPi, we did not detect any defect in RAD51 foci formation upon treatment with IR in CHTF18 KO cells (Fig. 1C). Importantly, an siBRCA2 control validated our RAD51 staining (Supplementary Fig. S1D).

### Treatment with PARPi leads to replication stress and ssDNA accumulation in CHTF18 KO cells

**Figure 2.**
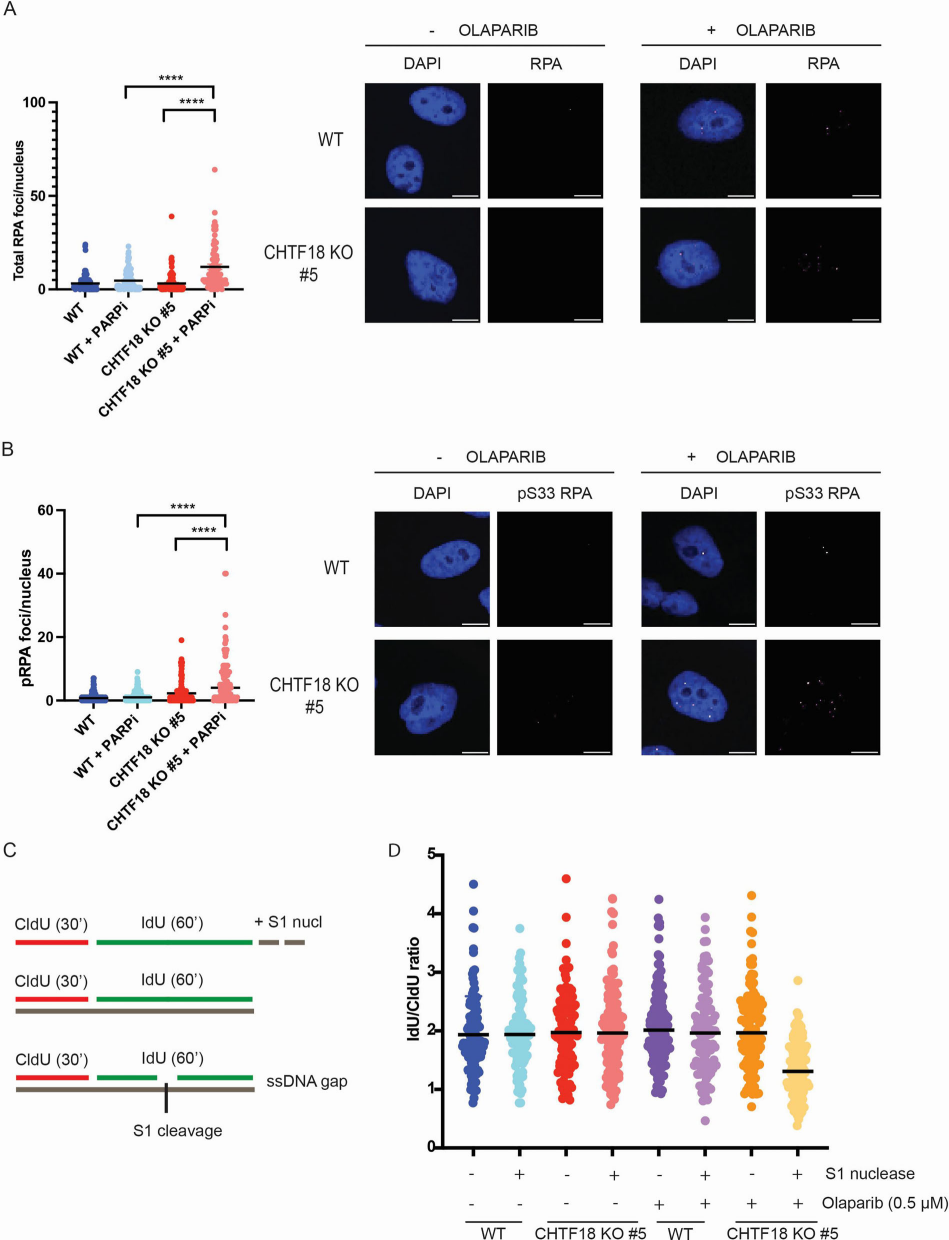
Treatment with PARPi induces replicative gap accumulation in CHTF18 KO cells. (**A**) Left: Bar-graphs showing the number of RPA foci/nucleus in the indicated cell lines treated or not with 0.5 μM Olaparib for 24 h. One hundred to one hundred fifty cells were analysed for condition. Right: Representative pictures from immunofluorescence staining for RPA in the indicated cell lines treated or not with Olaparib; unpaired *t*-test analysis, ****P < .0001. (**B**) Left: Bar-graphs showing the number of pS33 RPA foci/nucleus in the indicated cell lines treated or not with 0.5 μM Olaparib for 24 h. One hundred to one hundred fifty cells were analysed for condition. Right: Representative pictures from immunofluorescence staining for pS33 RPA in the indicated cell lines treated or not with Olaparib; unpaired *t*-test analysis, ****P < .0001. (**C**) Representative scheme of the CldU-IdU labelling strategy and S1 nuclease treatment performed to detect replicative gaps. (**D**) S1 nuclease assay: Bar-graphs showing IdU/CldU ratio, in the indicated cell lines treated or not with Olaparib, in the presence or absence of S1 nuclease. unpaired *t*-test analysis, ****P < .0001. One hundred to one hundred fifty fibers were analysed for condition.

An alternative possibility for the increased sensitivity of CHTF18 KO cells to PARPi is the accumulation of replicative DNA damage and replication stress. To analyse this possibility, we measured accumulation of single-stranded DNA (ssDNA) at replication fork by quantitative RPA chromatin immunofluorescence. Thus, we treated cells with 0.5 μM Olaparib for 24 h and fixed them with paraformaldeyde after pre-extraction of the nucleocytoplasm by CSK–Triton treatment. Strikingly, while CHTF18 KO cells showed minimal levels of RPA on chromatin in basal conditions, treatment with PARPi led to a significant increase of RPA levels in CHTF18 KO but not WT cells (Fig. 2A). To exclude a possible role for CHTF18 in checkpoint activation, we used quantitative chromatin immunofluorescence to measure the level of RPA phosphorylated on Ser33, a classic target of ATR and marker of replication stress. Consistently with replication stress and ssDNA accumulation, treatment with PARPi led to a remarkable increased phosphorylation of RPA on Ser33 in CHTF18 KO but not WT cells, also excluding a defective activation of the intra-S-phase checkpoint (Fig. 2B). Our RPA immunofluorescence analysis points to the accumulation of ssDNA at the replication forks in PARPi treated CHTF18 KO cells. We and others have recently shown that replicative gaps accumulate in POLE3–POLE4 KO cells upon treatment with PARPi [9, 10]. Furthermore, work form several laboratories have implicated replicative gaps in mediating PARPi sensitivity in a BRCA-mutant background [5, 12–15]. To investigate the post-replicative nature of ssDNA accumulation in CHTF18 KO cells, we then performed a DNA fiber assay in the presence and the absence of S1 nuclease (Fig. 2C) [11]. Strikingly, treatment with S1 nuclease led to a remarkable reduction of the IdU/CldU ratio in CHTF18 KO cells specifically upon treatment with PARPi pointing to the accumulation of replicative gaps in this condition (Fig. 2D). In summary, loss of CHTF18 triggers replicative gap accumulation and sensitivity to PARPi.

### CHTF18 KO cells are sensitive to transient depletion of both PRIMPOL and REV1

**Figure 3.**
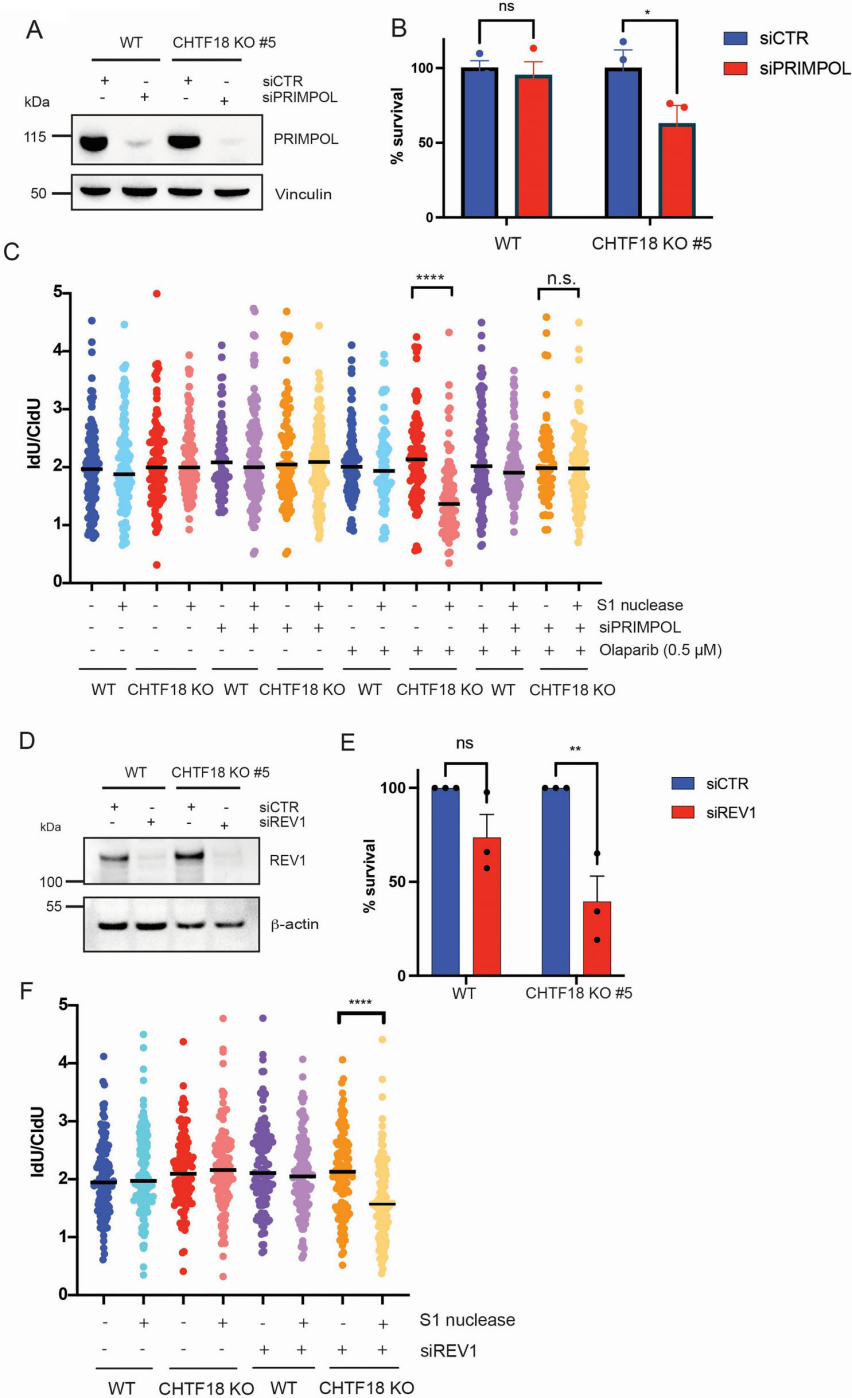
CHTF18 KO cells rely on PRIMPOL and REV1 for survival. (**A**) Western blot analysis of PRIMPOL in HTF WT, and CHTF18 KO cells transfected with siRNAs against PRIMPOL or Control (CTR). Vinculin was used for normalization. (**B**) Bar-graphs showing the percentage of clonogenic survival of HTF WT and CHTF18 KO cells transfected with siRNA against PRIMPOL or CTR. Results are normalized to siRNA CTR-transfected cells and were obtained from three independent biological experiments. (**C**) Bar-graphs showing IdU/CldU ratio, in the indicated cell lines transfected with siRNAs against PRIMPOL or control, treated or not with Olaparib, in the presence or absence of S1 nuclease; unpaired *t*-test analysis, ****P < .0001. One hundred to one hundred fifty fibers were analysed for condition. (**D**) Western blot analysis of REV1 in HTF WT and CHTF18 KO cells transfected with siRNAs against REV1 or CTR. β-ACTIN was used for normalization (**E**) Bar-graphs showing the percentage of clonogenic survival of HTF WT and CHTF18 KO cells transfected with siRNA against REV1 or CTR. Results are normalized to siRNA CTR-transfected cells and were obtained from three independent biological experiments. (**F**) Bar-graphs showing IdU/CldU ratio, in the indicated cell lines transfected with siRNAs against REV1 or control, in the presence or absence of S1 nuclease; unpaired *t*-test analysis, ****P < .0001. One hundred to one hundred fifty fibers were analysed for condition.

In mammalian cells, the restart of DNA replication at stalled leading strands requires repriming by PRIMPOL [16]. Given this fact and the accumulation of replicative gaps upon treatment with PARPi, we speculated that CHTF18 KO cells might be particularly dependent on PRIMPOL activity for survival. Consistently with this, transient silencing of PRIMPOL reduced survival of CHTF18 KO but not WT cells (Fig. 3A and B). Importantly and consistently with a re-priming phenomenon in PARPi-treated cells, silencing of PRIMPOL rescued replicative gap accumulation upon treatment with PARPi (Fig. 3C). Replicative gaps are filled-in predominantly by Translesion Synthesis (TLS) polymerases, such as REV1 [16, 17]. Consistently with a dependency on TLS for survival, silencing of REV1 reduced viability of CHTF18 KO but not WT cells (Fig. 3D and E). Finally, in agreement with constitutive activation of TLS in CHTF18 KO cells, silencing of REV1 led to the appearance of replicative gaps in the unchallenged condition (Fig. 3F). All together our data suggest that loss of CHTF18–RFC2/5 leads to re-priming events and gap filling by TLS polymerases.

### Sensitization to PARPi is refractory to the loss of 53BP1 in CHTF18 KO cells

**Figure 4.**
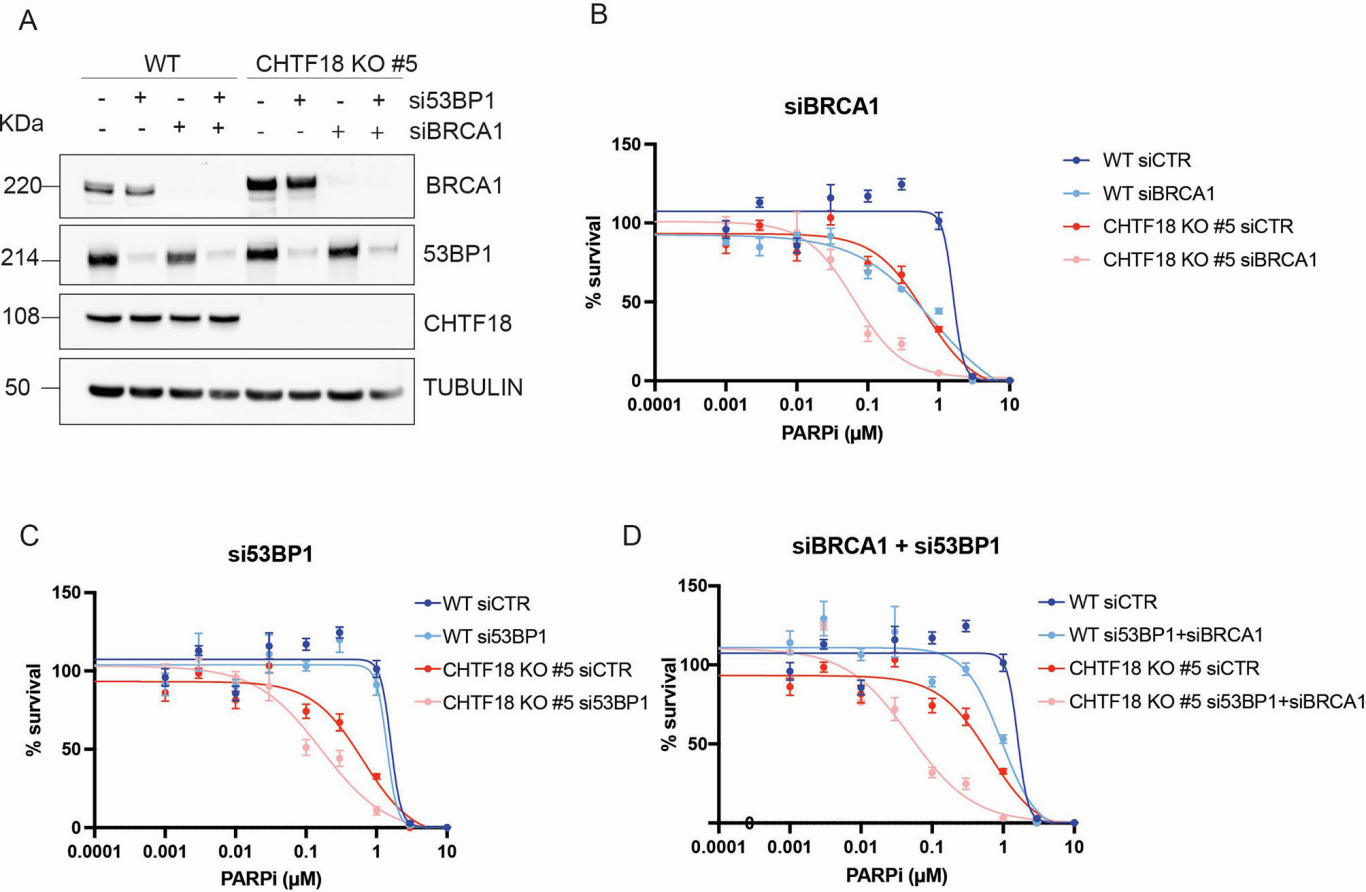
Sensitization to PARPi is resistant to loss of 53BP1 in CHTF18 KO cells. (**A**) Western blot analysis of BRCA1 and 53BP1 in HTF WT, and CHTF18 KO cells transfected with siRNAs against BRCA1, 53BP1, or CTR. Tubulin was used for normalization. (**B**) Quantification of clonogenic survival of CHTF18 KO and WT cells treated with Olaparib and transfected with siRNA against BRCA1 or CTR. Results were obtained from three independent biological experiments. (**C**) Quantification of clonogenic survival of CHTF18 KO and WT cells treated with Olaparib and transfected with siRNA against 53BP1. Results were obtained from three independent biological experiments. (**D**) Quantification of clonogenic survival of CHTF18 KO and WT cells treated with Olaparib and transfected with siRNA against both 53BP1 and BRCA1. Results were obtained from three independent biological experiments.

We and others have recently shown that sensitization to PARPi in POLE3–POLE4 KO cells is BRCA1 independent and resistant to deletion of 53BP1 [9, 10]. With this in mind, we analysed the interplay between CHTF18, BRCA1, and 53BP1 in sensitivity to PARPi (Fig. 4A). In agreement with what we observed in POLE3–POLE4 KO cells previously, silencing of BRCA1 further sensitized CHTF18 KO cells to PARPi, pointing to a non-epistatic mechanism of sensitization to PARPi (Fig. 4B). Loss of 53BP1 is a well-established mechanism of resistance to PARPi in BRCA1 null cell; whether loss of 53BP1 has a similar effect in CHTF18 KO cells remained to be investigated. With this in mind, we analysed cell viability of CHTF18 KO cells upon transient silencing of 53BP1, in the presence or absence of PARPi treatment. Strikingly, we discovered that unlike in the case of BRCA1-depleted cells, loss of 53BP1 does not lead to PARPi-resistance in CHTF18 KO cells (Fig. 4C and D). On the contrary, we observed further sensitization to Olaparib treatment, when 53BP1 was depleted in CHTF18 KO, but not WT cells. These data suggest that Polε-dependent replicative gaps are ‘resistant’ to loss of 53BP1, pointing to a novel potential strategy of sensitization of BRCA1 null cells to PARPi.

### PARP trapping at replicative gaps leads to sensitivity to PARPi

**Figure 5.**
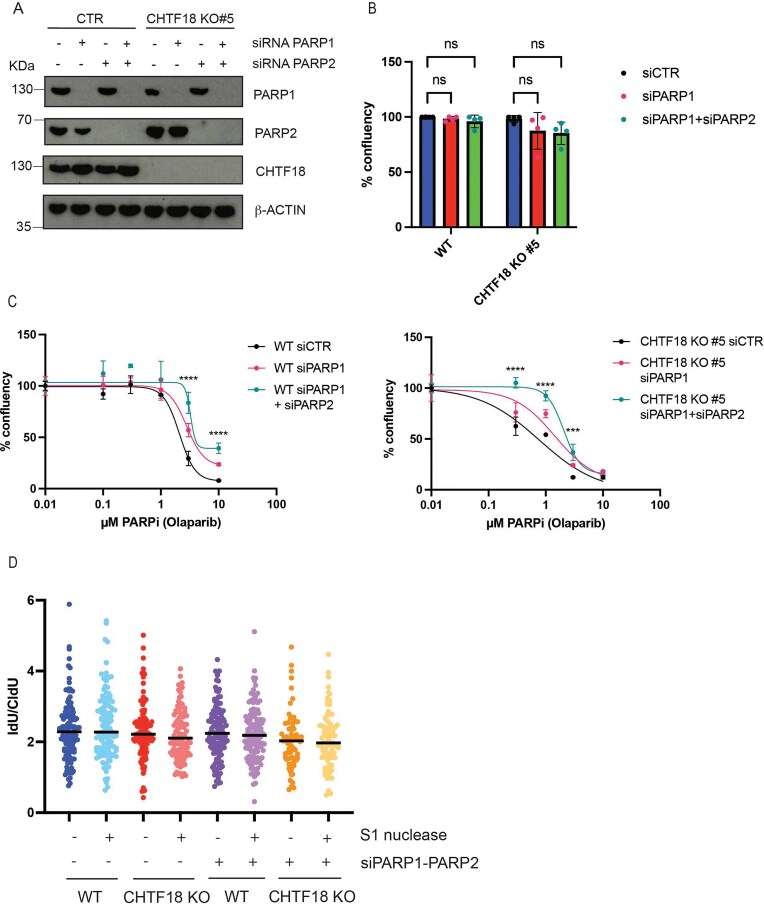
PARP trapping at replicative gaps causes PARPi sensitization. (**A**) Western blot analysis of PARP1 and PARP2 in HTF WT, and CHTF18 KO cells transfected with siRNAs against PARP1 and PARP2. β-ACTIN was used for normalization. (**B**) Bar-graphs showing % confluency in CHTF18 WT and KO cells transfected with siRNAs against PARP1 and PARP2. (**C**) Quantification of % confluency in the indicated cell lines treated or not with increasing concentration of PARPi (Olaparib). Results were obtained from three independent biological experiments. (**D**) S1 Nuclease assay: Bar-graphs showing IdU tract lenghts, in the indicated cell lines treated or not with S1 nuclease. One hundred to one hundred fifty fibers were analysed for condition.

Our data in CHTF18 KO and POLE3–POLE4 KO cells suggest that loss of Polε processivity is sensitizing cancer cells to PARPi. To dissect the role of PARP trapping in this phenomenon, we tested sensitivity of CHTF18 KO cells to Olaparib after PARP1 and PARP2 transient knockdown (Fig. 5A). Importantly CHTF18 KO cells were not hypersensitive to PARP1–PARP2 depletion in basal conditions (Fig. 5B). However transient silencing of PARP1 and PARP2 led to reduced sensitivity to Olaparib (Fig. 5C). A similar phenotype was observed in POLE3 and POLE4 KO cells CRISPR KO for PARP1 and PARP2, pointing to defective Polε activity (Supplementary Fig. S2A and B). These data suggest that replicative gap trapping has a crucial role in explaining sensitivity of cancer cells to PARPi. In order to further validate this possibility, we finally performed S1 nuclease assays in CHTF18 KO cells after silencing of PARP1 and PARP2 by siRNA. Consistently with a rescue of sensitivity to PARPi and the essential role of trapping, we observed the absence of replicative gaps in this condition (Fig. 5D). A similar phenotype was observed in POLE4–PARP1–PARP2 triple KO cells (Supplementary Fig. S2C). Overall these data point to PARP trapping as a crucial phenomenon underlying accumulation of replicative gaps and sensitivity to PARPi in cells compromised for Polε processivity.

## Discussion

The advent of PARPi has completely changed the treatment landscape of HR-deficient cancers [3]. Despite this, the development of resistance to PARPi is a major challenge in the clinical setting urging for novel approaches of sensitization to these compounds [6]. Importantly, the initial DNA lesion that sensitize cancer cells to PARPi remains highly debated [4]. Recently, replicative gaps emerged as a crucial driver of sensitization to PARPi in BRCA-mutant cancer cells [5].

Here, we initially show that replicative gap accumulation drives sensitization to PARPi upon loss of the CHTF18–RFC2/5 alternative clamp loader complex. Genetic deletion of CHTF18 causes specifically hyper-sensitivity to ATR and PARPi, pointing to a replication-dependent mechanism. Consistently with this, treatment with Olaparib triggered RPA phosphorylation on Ser 33, a classic marker of replication stress, and the accumulation of replicative gaps as visualized by S1 nuclease and DNA fiber staining. Importantly, and similarly to POLE3–POLE4 KO cells, CHTF18 KO cells are proficient in RAD51 foci formation, excluding a defect in HR. CHTF18 KO cells are also not hypersensitive to Hydroxyurea, Mitomycin C, or Pyridostatin, excluding unspecific replicative defects. We have recently shown that CHTF18–RFC2/5 and POLE3–POLE4 represent two tiers of regulation of Polε processivity. Thus, leading strand-specific loading of PCNA by the CHTF18–RFC2/5 complex acts in concert with dsDNA gripping by POLE3–POLE4 to sustain continuous and uninterrupted DNA synthesis by Polε [8]. Our data in CHTF18 and POLE3–POLE4 KO cells suggest that loss of Polε processivity is driving sensitization to PARPi. CHTF18 null cells are sensitive to transient knockdown of PRIMPOL, suggesting that repriming downstream Polε stalling events is crucial in CHTF18-null cells to sustain normal DNA replication rates and genome stability. Consistently with this, CHTF18 KO cells were also relying on gap filling by the REV1 TLS Polymerase for survival, and transient silencing of REV1 led to replicative gap accumulation in CHTF18 KO cells.

By performing transient silencing experiments in CHTF18 KO cells treated with Olaparib, we defined loss of PARP1 and PARP2 as drivers of resistance to PARPi in this genetic context. Of note, CHTF18 KO cells were more sensitive than WT cells to PARPi at high doses, even upon transient silencing of PARP1 and PARP2. This might reflects higher sensitivity to off-target effects of PARPi or be the results of partial silencing by siRNAs of PARP1-2. We speculate that loss of Polε processivity leads to the formation of transient replicative gaps that are rapidly filled in by TLS polymerases and/or by a switch to DNA polymerase delta. Treatment with PARPi leads to trapping of PARP1 and PARP2 at replicative gaps precluding their spontaneous repair. Consistently with this, we did not observe replicative gaps upon silencing of PARP1–PARP2 in CHTF18 KO cells.

As a limitation, our study was prevalently performed in HeLa cells. Thus, whether silencing of CHTF18–RFC2/5 sensitizes BRCA-mutant cancer cells to PARPi remains to be investigated. Despite this, transient silencing of CHTF18 by siRNA led to sensitization to PARPi also in eHAP cells, excluding a cell line specific phenomenon.

Importantly, CHTF18 is an AAA + ATPase, that can be targeted by small molecule inhibitors (https://depmap.org/portal/gene/CHTF18?tab=overview). Indeed, recently, small molecule inhibitors targeting ATPase activities of POLQ [18] or WRN helicase [19, 20] were shown to be effective in specific killing of HR-deficient or microsatellite instable cancer cells, and are currently in clinical trials. The fact that CHTF18 loss further sensitizes BRCA1-deficient cancer cells to PARPi in a manner that is refractory to loss of 53BP1, and that, unlike the catalytic activity of Polε, CHTF18 is not required for viability, makes CHTF18 a promising target in the treatment of cancers resistant to PARPi.

In summary, our work highlights trapping of replicative gaps as a driver of sensitization to PARPi and suggests that targeting Polε processivity and/or the CHTF18/RFC2-5 complex might represent a novel strategy to address PARPi resistance in the clinic.

## Supplementary Material

zcag013_Supplemental_File

## Data Availability

The data underlying this article are available in the article and in its online supplementary material.
