# Supplementary material for: Loss of CHTF18–RFC2/5 leads to replicative gaps and sensitivity to PARP inhibitors
Source: NAR Cancer. 2026 Jun 3;8(2):zcag013. doi: 10.1093/narcan/zcag013 (PMC13231162; doi:10.1093/narcan/zcag013)
Supplement: zcag013_Supplemental_File [file zcag013_supplemental_file.pdf]

Supplemental information

Loss of CHTF18-RFC2/5 leads to replicative gaps and sensitivity to PARP inhibitors

Lauryn Buckley-Benbow[1], Meryem Ozgencil[1], Alessia Tardocchi[1,2], Alessandro Agnarelli and Roberto Bellelli[1#]

Figure S1

A

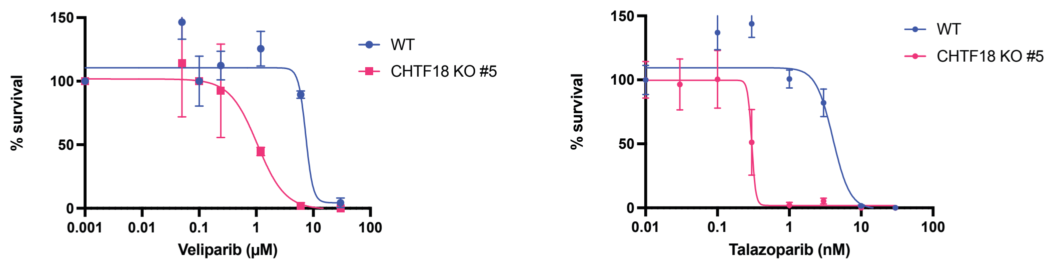

B

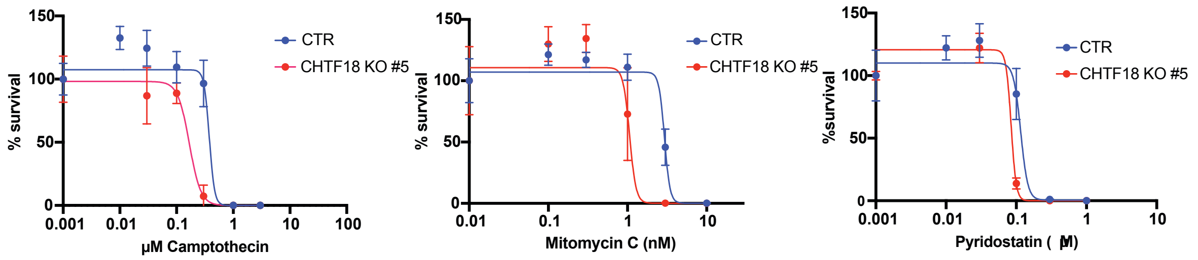

C

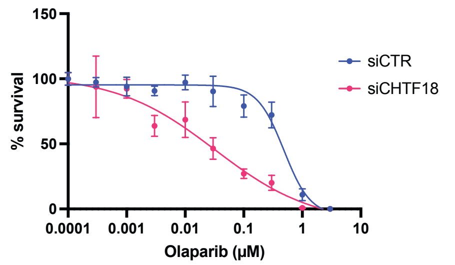

D

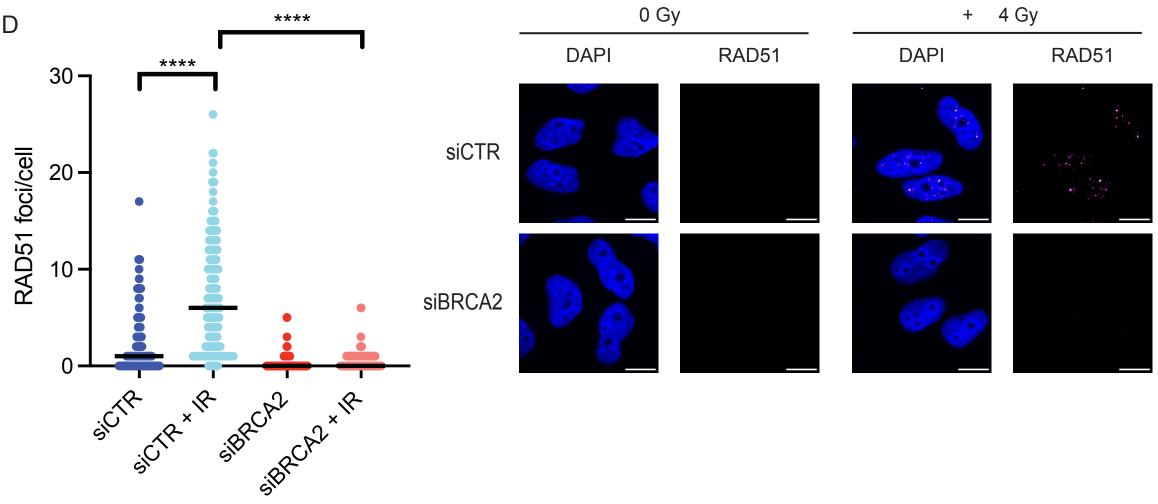

Figure S1.

A) Quantification of clonogenic survival of CHTF18 KO and WT cells treated with Veliparib or Talazoparib. B) Quantification of clonogenic survival of CHTF18 KO and WT cells treated with Camptothecin, Mitomycin C or Pyridostatin. C) Quantification of clonogenic survival of eHAP cells treated with Olaparib and transfected with siRNA against CHTF18 or CTR. D) Left: Bar-graphs showing the number of RAD51 foci/nucleus in the indicated cell lines treated or not with 4 Gy IR. 150-200 cells were analysed for condition. Right: Representative pictures from immunofluorescence staining for RAD51 in the indicated cell lines treated or not with 4 Gy IR; unpaired t-test analysis, **** p<0.0001.

Figure S2

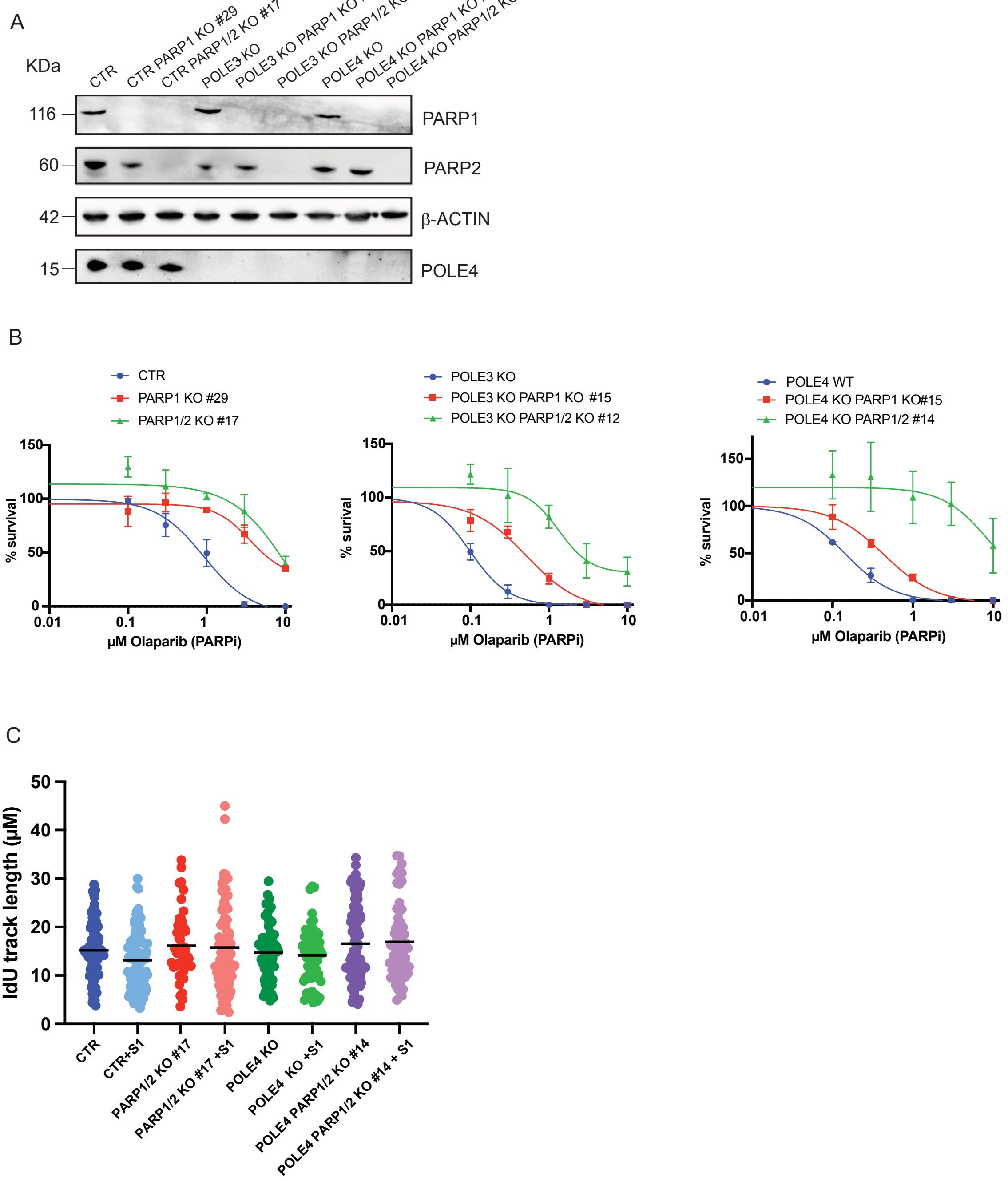
A
KDa
CTR
CTR PARP1 KO #29
CTR PARP1/2 KO #17
POLE3 KO
POLE3 KO PARP1 KO #15
POLE3 KO PARP1/2 KO #12
POLE4 KO
POLE4 KO PARP1 KO #15
POLE4 KO PARP1/2 KO #14
116
60
42
15
PARP1
PARP2
β-ACTIN
POLE4
B
CTR
PARP1 KO #29
PARP1/2 KO #17
% survival
µM Olaparib (PARPi)
POLE3 KO
POLE3 KO PARP1 KO #15
POLE3 KO PARP1/2 KO #12
% surival
POLE4 WT
POLE4 KO PARP1 KO#15
POLE4 KO PARP1/2 #14
150
100
50
0
0.01
0.1
1
10
C
IdU track length (µM)
50
40
30
20
10
0
CTR
CTR+S1
PARP1/2 KO #17
PARP1/2 KO #17 +S1
POLE4 KO
POLE4 KO +S1
POLE4 PARP1/2 KO #14
POLE4 PARP1/2 KO #14 + S1

Figure S2.

A) Western blot analysis of PARP1 and PARP2 in the indicated cell lines. β-Actin was used for normalization. B) Quantification of clonogenic survival in the indicated cell lines treated or not with increasing concentration of PARPi (Olaparib). Results were obtained from 3 independent biological experiments. C) S1 Nuclease assay: Bar-graphs showing IdU tract lenghts, in the indicated cell lines treated or not with S1 nuclease.
